# A rapid ammonium fluoride method to determine the oxygen isotope ratio of available phosphorus in tropical soils

**DOI:** 10.1002/rcm.8647

**Published:** 2020-02-21

**Authors:** Verena Pfahler, Aleksandra Bielnicka, Andrew C. Smith, Steven J. Granger, Martin S.A. Blackwell, Benjamin L. Turner

**Affiliations:** ^1^ Sustainable Agriculture Sciences Rothamsted Research North Wyke Okehampton EX20 2SB UK; ^2^ Smithsonian Tropical Research Institute Apartado 0843‐03092 Balboa Ancon Republic of Panama; ^3^ NERC Isotope Geoscience Laboratory British Geological Survey Nottingham NG12 5GG UK

## Abstract

**Rationale:**

The isotopic composition of oxygen bound to phosphorus (δ^18^O_P_ value) offers an opportunity to gain insight into P cycling mechanisms. However, there is little information for tropical forest soils, which presents a challenge for δ^18^O_P_ measurements due to low available P concentrations. Here we report the use of a rapid ammonium fluoride extraction method (Bray‐1) as an alternative to the widely used anion‐exchange membrane (AEM) method for quantification of δ^18^O_P_ values of available P in tropical forest soils.

**Methods:**

We compared P concentrations and δ^18^O_P_ values of available and microbial P determined by AEM and Bray‐1 extraction for a series of tropical forest soils from Panama spanning a steep P gradient. This involved an assessment of the influence of extraction conditions, including temperature, extraction time, fumigation time and solution‐to‐soil ratio, on P concentrations and isotope ratios.

**Results:**

Depending on the extraction conditions, Bray‐1 P concentrations ranged from 0.2 to 66.3 mg P kg^−1^ across the soils. Extraction time and temperature had only minor effects on Bray‐1 P, but concentrations increased markedly as the solution‐to‐soil ratio increased. In contrast, extraction conditions did not affect Bray‐1 δ^18^O_P_ values, indicating that Bray‐1 provides a robust measure of the isotopic composition of available soil P. For a relatively high P soil, available and fumigation‐released (microbial) δ^18^O_P_ values determined by Bray‐1 extraction (20‰ and 16‰, respectively) were higher than those determined by the AEM method (18‰ and 12‰, respectively), which we attribute to slightly different P pools extracted by the two methods and/or differences resulting from the longer extraction time needed for the AEM method.

**Conclusions:**

The short extraction time, insensitivity to extraction conditions and smaller mass of soil required to extract sufficient P for isotopic analysis make Bray‐1extraction a suitable alternative to the AEM method for the determination of δ^18^O_P_ values of available P in tropical soils.

## 1 INTRODUCTION

Tropical forest soils sustain a large net primary production despite low phosphorus (P) availability.^1^ Given the importance of understanding how tropical forests will react to future environmental change, and the role of soil P in regulating these responses, there is an urgent need to better understand P cycling in tropical forest soils.^2^ This requires the development of novel procedures that can provide information on the dynamics of P in the soil–plant–microbe continuum.

A promising technique for the investigation of soil P cycling involves the determination of the ^18^O:^16^O ratio of oxygen (O) bound to P (δ^18^O_P_ value).^3^, ^4^, ^5^, ^6^ The δ^18^O_P_ technique has been used to investigate the importance of microorganisms for P cycling^7^ and can provide information about hydrolysis by phosphatase enzymes^8^, ^9^ and the origin of P inputs into aquatic systems.^10^, ^11^ However, information on δ^18^O_P_ values in tropical forest soils remains scarce, despite the importance of P in the ecology of this hyper diverse biome.^1^ Indeed, the only study so far involved the quantification of δ^18^O_P_ values in soils from litter and fertilization experiments in Panama, which suggested the importance of microorganisms for P cycling in lowland tropical soils.^12^

The main method for quantifying the δ^18^O_P_ values of available P is extraction via an anion‐exchange membrane (AEM).^13^ However, a number of potential issues limit the use of the AEM method for tropical soils, including the low available P concentrations (often <1 mg P kg^−1^)^14^ and enzymatic activity during the extraction and storage of the soil samples leading to O exchange during the extraction and storage. To address the problem of low P concentrations, Weiner et al^13^ upscaled the conventional AEM extraction method to 100 g dried soil and 5 L of water. However, to obtain the required amount of approximately 0.8 mg P for the determination of the δ^18^O_P_ values,^13^ approximately 1 kg dried soil and 50 L of water would be necessary for tropical soils.^12^ In addition, the relatively long extraction time for the AEM method might influence results for δ^18^O_P_ values, particularly for the determination of δ^18^O_P_ values in microbial biomass, if enzymatic activity leads to hydrolysis of organic P during the extraction. It is therefore recommended that AEM extractions for δ^18^O_P_ measurement be performed at 4°C,^15^ which presents an additional limitation on the procedure.

Several alternative extraction procedures exist for soil available P that might be suitable for the determination of δ^18^O_P_ values, including extraction in water and sodium bicarbonate.^16^, ^17^ Water extracts, however, can contain considerable concentrations of fine clays, which are difficult to remove by filtration and interfere with analysis, and water‐extractable P concentrations in tropical soils are usually even lower than in AEM extracts.^18^ In contrast, P concentrations in sodium bicarbonate extracts are usually greater than in water extracts, but the high solution pH, carbonate and salt concentration could lead to problems during the purification of P for δ^18^O_P_ determination. Degassing prior to the purification and precipitation of brucite is recommended to further clean the extracts.^19^, ^20^ In addition, sodium bicarbonate extracts are slightly alkaline, and can therefore extract a considerable amount of organic P. The purification protocol for the δ^18^O_P_ determination only targets inorganic P, but extracted organic P could be hydrolysed under the acidic conditions of the colorimetric assay of orthophosphate.^21^ As the orthophosphate concentrations are used to calculate the δ^18^O_P_ values of microbial P, hydrolysis of organic P might lead to erroneous results.

An alternative procedure involves the extraction of available P in acidic ammonium fluoride (Bray‐1 extraction; 30 mM NH_4_F + 25 mM HCl).^22^ The method is appropriate for tropical soils because it is designed to extract P from acidic soils and extracts little organic P (the extraction is conducted at pH 2.5).^23^ The NH_4_F prevents re‐adsorption of P onto metal oxides, which are abundant in strongly weathered tropical soils. Importantly, the extraction time for the Bray‐1 method is considerably shorter than for the AEM method (minutes compared with hours), which favours the accurate determination of the δ^18^O_P_ values because enzymatic activity during the extraction could lead to changes in the δ^18^O_P_ value. Indeed, the method also appears suitable for δ^18^O_P_ determination, because McLaughlin et al^24^ purified Bray‐1 soil extracts and precipitated Ag_3_PO_4_, although they did not provide information about potential artefacts or interferences during the purification.

We therefore investigated whether the Bray‐1extraction could provide a rapid alternative to the AEM method for determining the δ^18^O_P_ values of available and microbial P in tropical soils. To do this, we assessed whether δ^18^O_P_ values and concentrations of available P determined in Bray‐1 extracts were altered by extraction conditions, including solution‐to‐soil ratio, extraction temperature and time. We then used different fumigation times to test how this affected the δ^18^O_P_ values of microbial P. Finally, we compared the δ^18^O_P_ values of Bray extracts with those obtained by the AEM method.

## 2 EXPERIMENTAL

### 2.1 Soil sampling and analysis

Soils were collected from six locations under lowland tropical forest in central Panama in January and February 2017 during the early dry season. The locations are part of a broader network of forest census sites; detailed information on the locations, the tree community and soils is published elsewhere.^14^, ^25^, ^26^, ^27^ The sample sites were chosen to represent a range of P concentrations, soil taxonomy and parent materials (Table 1).

**Table 1:** Site description and soil properties

Site	Coordinates	Parent material	Soil taxonomy	pH (water)	LOI (%)	Total P (mg P/kg)	Resin P (mg P/kg)
Madden Dam	9.211°N, 79.600°W	Calcareous sandstone	Mollisols	6.6	25.2	1542	22.8
Plantation Road	9.090°N, 79.653°W	Andesite	Inceptisols (provisional)	6.4	18.3	1127	13.3
Plot 05	9.157°N, 79.752°W	Marine sediments	Alfisols	6.1	16.6	428	1.9
Plot 15	9.162°N, 79.745°W	Marine sediments	Alfisols	5.4	10.5	319	1.2
Plot 07	9.161°N, 79.743°W	Marine sediments	Oxisols	4.2	12.4	282	1.4
Plot 08	9.168°N, 79.746°W	Basalt	Oxisols	4.4	13.3	264	0.8

Soil samples were taken from the upper 10 cm of the soil, sieved (<2 mm) fresh, stored at 4°C and extracted within 2 weeks of sampling.

### 2.2 Extractions

All extractions involved fresh soils, and solution‐to‐soil ratios were based on fresh weights and not dry weights. However, data is reported on the basis of oven‐dry soil. Phosphorus concentrations in the extracts are referred to as P_unf_ (P in unfumigated extracts) and P_fum_ (P in liquid (hexanol) or gaseous (chloroform) fumigated extracts). Based on pre‐tests, we decided not to replicate the extractions for the determination of P concentrations, because the error associated with replicate extractions was <5%.

For AEM extractions we followed the protocol of Turner and Romero.^28^ In brief, 10 g fresh soil, 80 mL ultrapure (18.2 MΩ) water and five resin strips (1.5 × 4 cm) were used (unfumigated extracts). Fumigated extracts received an additional 1 mL hexanol. To test for a temperature effect on P concentrations, the samples were shaken overnight at 22°C or 4°C. On the following day, the resin strips were removed, cleaned with ultrapure water and eluted for 1 h in 50 mL 0.25 M sulfuric acid (H_2_SO_4_).

Table 2 summarizes the different extraction characteristics tested for the Bray‐1method (fumigation with CHCl_3_ vapor).^29^ We tested the effect of fumigation time by using three different times. Two were based on literature reports: Oberson et al^23^ (75 min) and Brookes et al^29^ (24 h = 1440 min). The third (15 min) was chosen to provide sufficient time to lyse microbial cells, but minimize the time to hydrolyse intracellular organic P, which could influence the δ^18^O_P_ values.

**Table 2:** Summary of the different extraction characteristics for the Bray‐1 method used for unfumigated and fumigated samples

Unfumigated	Fumigated
Solution‐to‐soil ratio 1, 2, 3, 5, 7, 8, 10, 15, 20, 25, 30, 40, 50, 100	Solution‐to‐soilratio 10
Extraction temperature 4°C, 22°C	Extraction temperature 22°C
Extraction time (min) 5, 15, 30, 60, 960	Extraction time (min) 5, 15
	Fumigation time (min) 15, 75, 1440

After extraction, samples were centrifuged (3000 *g*, 15 min) and filtered through Whatman 42 filter papers. The P concentrations in all extracts were determined by molybdate colorimetry.^30^ Phosphorus released by fumigation (fumigation‐released P) was calculated as the difference between the concentrations of the fumigated and unfumigated extracts. We did not determine P recovery to correct for P adsorption during the extractions, as the recovery of P spikes is not comparable with the recovery of microbial P released during chloroform fumigation in acidic soils.^31^

For the δ^18^O_P_ values of AEM P_unf_ and P_fum_, we used the same solution‐to‐soil ratio as for the determination of the P concentrations but, depending on the P concentrations, we used 200–600g fresh soil for AEM P_unf_ and 100–200 g fresh soil for AEM P_fum_ (instead of the normal 10 g) to obtain sufficient P for analysis.

Soils from Plantation Road and Madden Dam were used for the determination of the δ^18^O_P_ values of Bray‐1 P_unf_ and P_fum_ using a solution‐to‐soil ratio of 10, extraction time of 5 min and an extraction temperature of 22°C. Those two soils were chosen for their contrasting properties, including P concentrations, organic carbon content and soil taxonomic class (Table 1). In addition, the soil from Madden Dam was used to investigate the effect of the solution‐to‐soil ratio and extraction temperature on the δ^18^O_P_ value of Bray‐1 P_unf_. The solution‐to‐soil ratios were: 5, 10 and 50. A ratio of 10 is the standard solution‐to‐soil ratio used for Bray‐1 extractions.^32^ The other two ratios were a compromise between amount of P extracted and volume of Bray‐1 solution needed. Extractions of soil from Madden Dam were carried out with ^18^O‐labelled and unlabelled Bray‐1 solutions to account for any hydrolysis of organic and/or condensed P during the extractions and subsequent O exchange between phosphate and the solution.^7^ Soil from Madden Dam was chosen as organic and the condensed P concentrations are amongst the highest found so far in tropical soils.^33^ If there is no noteworthy O exchange in the case of Madden Dam, we assume that this would also be the case for soils with lower organic/condensed P concentrations.

### 2.3 Measurement of oxygen isotope ratio

The AEM and Bray‐1 extracts were purified following Tamburini et al,^34^ but with the addition of 1 mL concentrated H_2_SO_4_ during the ammonium phosphomolybdate (APM) step to facilitate the precipitation of the crystals.^35^ Measurement of the δ^18^O_P_ values was undertaken by weighing approx. 300 μg of Ag_3_PO_4_ into a silver capsule to which a small amount of fine glassy carbon powder was added to aid combustion.^34^ The sample was converted into carbon monoxide at 1400°C in a thermal conversion elemental analyzer (Thermo Fisher Scientific Inc., Bremen, Germany), with the resultant CO passing through a gas chromatography (GC) column into a Delta + XL isotope ratio mass spectrometer (Thermo Fisher Scientific Inc.) via a ConFlo III interface (Thermo Fisher Scientific Inc.). The δ^18^O_P_ value was calculated by comparison with the internal Ag_3_PO_4_ laboratory standard, ALFA‐1 (ALFA‐1 = δ^18^O VSMOW value of 14.2‰). In the absence of an international Ag_3_PO_4_ reference material, we derived this value for ALFA‐1 by comparison with the Ag_3_PO_4_ standard ‘B2207’ (Elemental Microanalysis Ltd, Okehampton, UK), which has been measured in an inter‐laboratory comparison study to have a δ^18^O value of 21.7‰ vs VSMOW. Samples were run in duplicates, with a typical precision of σ ≤0.3‰, while the standard material B2207 had a typical precision across runs of σ ≤0.5‰. δ^18^O_P_ values of the samples were rejected if the O yield of the sample differed by >10% from the O yield of the reference. The δ^18^O values of the^18^O‐labelled and unlabelled Bray‐1 solutions were determined on an Aquaprep inlet device (Isoprime Ltd, Cheadle, UK) coupled to an Isoprime 100 dual‐inlet isotope ratio mass spectrometer through a process of headspace CO_2_ equilibration with water samples. The isotope ratios are reported as δ^18^O values vs VSMOW, based on comparison with laboratory standards calibrated against IAEA standards, VSMOW and SLAP, with analytical precision typically σ ≤0.05‰.

The oxygen isotope ratios are reported in the conventional delta notation:

(1) $$\delta \left({}^{18}O\right)=\left(\frac{R_{\text{sample}}}{R_{\text{standard}}}-1\right),$$

where *R* = ^18^O/^16^O and *R*_standard_ is the VSMOW.

## 3 CALCULATIONS

The effect of the solution‐to‐soil ratio and extraction temperature on the Bray‐1 P_unf_ concentrations was tested using a two‐way analysis of variance (ANOVA). A t‐test (α = 0.05) was used to check if δ^18^O_P_ values differed depending on whether ^18^O‐labelled or unlabelled Bray‐1 solutions were used. Based on the result of the t‐test, the δ^18^O_P_ values obtained with^18^O‐labelled and unlabelled Bray‐1 solutions were considered as replicates for the other treatments (solution‐to‐soil ratio, extraction temperature and fumigation time) and not as a separate set of samples. One‐way ANOVAs followed by Tukey's HSD tests (α = 0.05) was then used to test the effect of solution‐to‐soil ratio, extraction temperature and fumigation time on the δ^18^O_P_ values. In cases where the requirements for ANOVA were not fulfilled, a Kruskal Wallis rank sum test was used to evaluate the data. This was only the case when testing the effect of the solution‐to‐soil ratio on the δ^18^O_P_ values. The δ^18^O_P_ values of microbial P were calculated via mass balance using concentrations and the δ^18^O_P_ values of the P_unf_ and P_fum_ extracts.^15^ All statistical analyses were performed with the program R.^36^

## 4 RESULTS

### 4.1 Phosphorus concentrations

**Figure 1:**
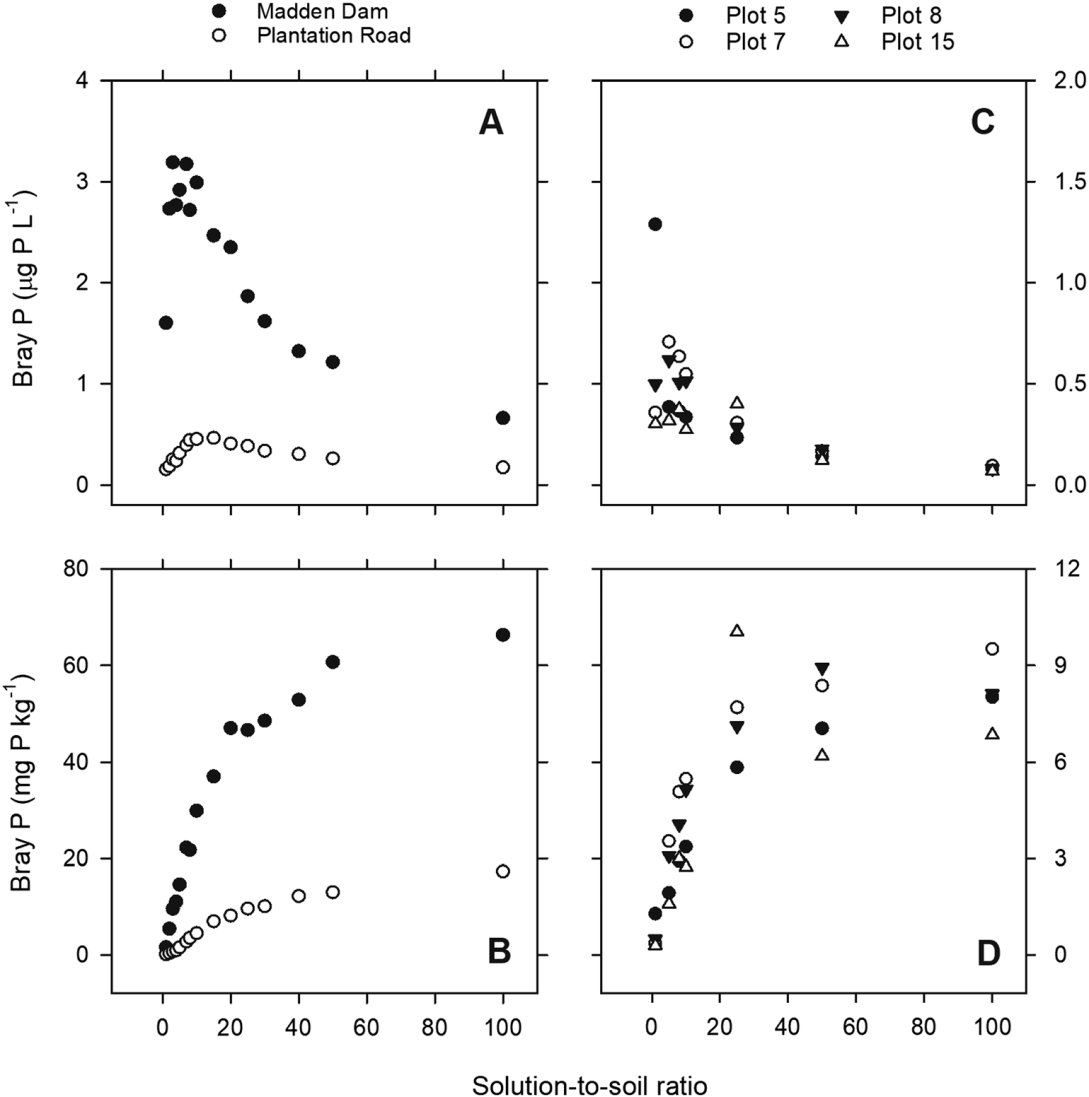
Effect of the solution‐to‐soil ratio on the phosphorus (P) concentrations (in µg P L^−1^ Bray‐1 solution (A and C) and mg P kg^−1^ soil (B and D) of unfumigated Bray‐1 extracts for soils from Madden Dam and Plantation road (A and B) and plots 5, 7, 8, and 15 (C and D)

With increasing solution‐to‐soil ratio the P_unf_ concentration in the Bray‐1 extracts increased between 6‐ and 112‐fold, depending on the soil (Figure 1). The largest proportional increase in P_unf_ concentrations was for Plantation Road, which has high total P concentrations, and the lowest for Plot 5, which contained a relatively low total P concentration (Table 1). The largest absolute increase in P_unf_ concentration was observed for Madden Dam (Figure 1).

**Figure 2:**
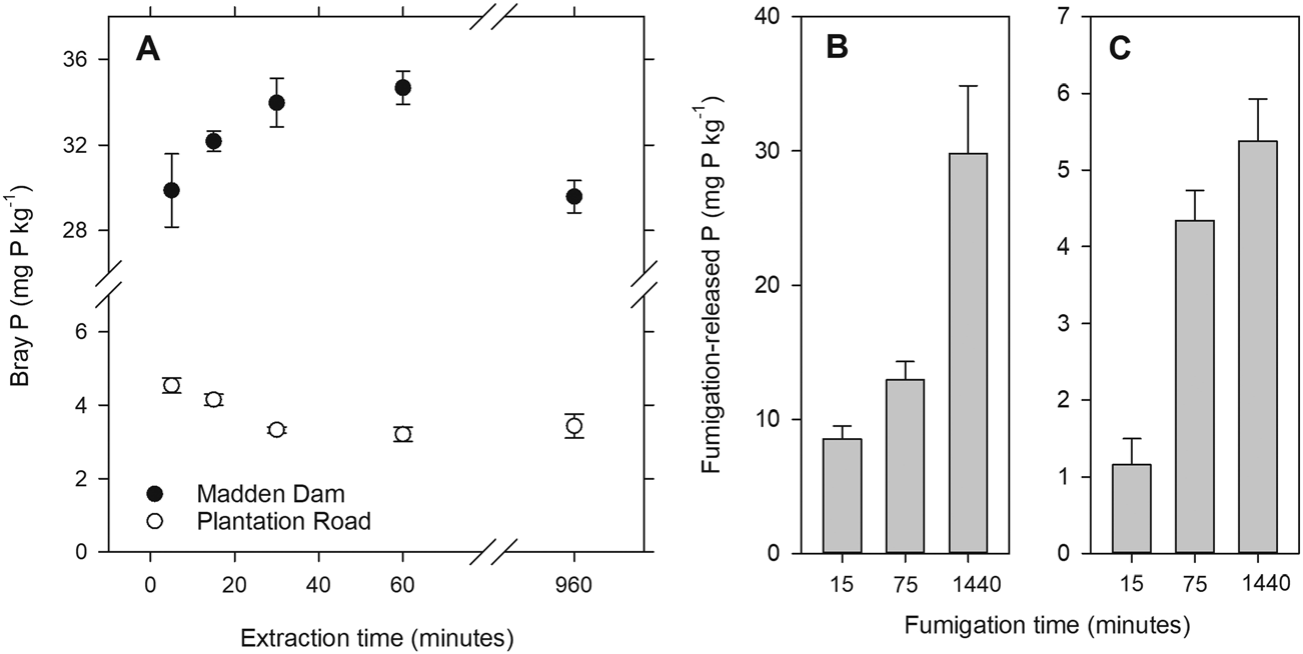
Effect of the extraction time on the phosphorus (P) concentrations (in mg P kg^−1^ soil) of unfumigated Bray‐1 extracts for soils from Madden Dam and Plantation Road (A). Effect of fumigation time on the amount of P released during the fumigation (calculated as difference between P_fum_ and P_unf_; in mg P kg^−1^ soil) for soil from Madden Dam (B) and Plantation Road (C)

With increasing extraction time, the Bray‐1 P_unf_ concentrations for Madden Dam soil first increased, but then decreased between 60 and 960 min, presumably due to resorption during the extraction. For the Plantation Road soil, Bray‐1 P_unf_ decreased with extraction time. Increasing the fumigation time up to 24 h increased the Bray‐1 fumigation‐released P for Madden Dam and Plantation Road (Figures 2B and 2C).

Extraction at 4°C compared with 22°C increased the Bray‐1 P_unf_ concentrations slightly, but significantly (*p* < 0.05), for Madden Dam, but not for Plantation Road (*p* > 0.1) (Table 3).

**Table 3:** Concentrations of phosphorus (P) (in mg kg^−1^ soil) extracted with anion exchange membrane (AEM) and Bray‐1 solution without (P_unf_) and with addition (P_fum_) of hexanol (AEM) or chloroform (Bray‐1)

Site	Extraction method	Solution‐to‐soil ratio	P_unf_		P_fum_	
			Extraction temperature			
			4°C	22°C	4°C	22°C
Plantation Road	AEM		1.6	1.5	9.7	9.7
	Bray‐1	1	0.3	0.2		
		5	1.4	1.6		
		8	3.4	3.6		
		10	4.8	4.5		8.8
		25	10.7	9.6		
		50	14.8	13.0		
		100	13.7	17.3		
Madden Dam	AEM		12.2	22.7	57.4	89.3
	Bray‐1	1	3.5	1.6		
		5	22.5	14.6		
		8	34.0	21.7		
		10	37.8	29.9		59.4
		25	59.0	46.6		
		50	71.2	60.6		
		100	69.5	66.3		

The extraction temperature did not affect the AEM P_unf_ concentrations for Plantation Road, but the AEM P_unf_ and P_fum_ concentrations increased by a factor of 1.6 for Madden Dam when extracted at 22°C compared with 4°C (Table 3).

### 4.2 δ^18^O_P_ values

The δ^18^O_P_ values of AEM P_unf_ and P_fum_ for Plantation Road were 16.5‰ and 14.3‰, respectively, while for Madden Dam the values were 18.0‰ and 13.5‰, respectively. The corresponding δ^18^O_P_ values of microbial P were 13.9‰ for Plantation Road and 12.3‰ for Madden Dam.

The δ^18^O_P_ values for Bray‐1 P_unf_ and P_fum_ for Madden Dam are shown in Table 4. The δ^18^O_P_ values of Bray‐1 P_unf_ and P_fum_ were not affected by using^18^O‐labelled and unlabelled Bray‐1 solutions, indicating that there was no O exchange between phosphate and the Bray‐1 solution during the extraction (t‐test, *p‐*value > 0.5). In addition, the δ^18^O_P_ values of Bray‐1 P were not affected significantly by extraction temperature (P_unf_; *p‐*value >0.1), soil‐to‐solution ratio (P_unf_; *p‐*value >0.1) or increasing fumigation time (P_fum_; *p‐*value >0.1).

**Table 4:** δ^18^O_P_ values of fumigated and unfumigated Bray‐1 extracts from Madden Dam using^18^O‐labelled and unlabelled Bray‐1 solution. δ^18^O_P_ values are given in ‰, numbers in brackets are standard deviations. *n* = 2 for the different treatments, where no standard deviation is given *n* = 1. Nd = not determined

	Solution‐to‐soil ratio/fumigation time	22°C			4 °C		
		Labelled	Unlabelled	Average	Labelled	Unlabelled	Average
Unfumigated*	5	19.3	18.6	18.9 (0.5)	20.4 (0.3)	20.7 (3.2)	20.5 (1.8)
	10	Nd	21.0 (0.2)	21.0 (0.2)	21.6	22.8	22.2 (0.9)
	50	20.0 (0.1)	19.2 (0.6)	19.6 (0.5)	19.5	19.6	19.6 (0.1)
Fumigated†	15 min	21.0 (1.3)	19.6 (1.0)	20.2 (1.3)	Nd	Nd	
	75 min	20.6 (1.6)	20.3 (0.9)	20.4 (1.1)	Nd	Nd	
	1440 min	19.1 (0.1)	18.9 (0.1)	19.0 (0.2)	Nd	Nd	

* Unfumigated samples were extracted for 5 min (22°C) and 15 min (4°C), respectively.

† Fumigated samples were extracted for 5 min.

Based on the average value of Bray‐1 P_unf_ (22°C, solution‐to‐soil ratio of 10) and the average values of Bray‐1 P_fum_, the calculated δ^18^O_P_ values of microbial P were as follows: 16.8‰ (fumigation time 15 min), 19.2‰ (75 min) and 16.5‰ (1440 min). For Plantation Road, δ^18^O_P_ values of Bray‐1 could only be determined for P_fum_; these values were 20.1‰ (fumigation time 15 min), 20.2‰ (75 min) and 19.9‰ (1440 min).

## 5 DISCUSSION

### 5.1 The δ^18^O_P_ values of Bray‐1 extracts and the influence of extraction conditions

Using^18^O‐labelled and unlabelled Bray‐1 solutions revealed that there was no O‐exchange during the extraction, regardless of whether or not the samples were fumigated. This means that no detectable hydrolysis of organic and/or condensed phosphate occurred during the extraction with Bray‐1 solution, which thus preserves the isotopic ratio of the target available P pool. Extraction conditions such as the solution‐to‐soil ratio are known to influence the amount of P extracted from soils, but did not affect the δ^18^O_P_ values of Bray‐1 P_unf_, despite marked changes in P concentrations depending on the extraction conditions. This indicates that the soil P pool extracted via Bray‐1 remains the same, despite the increasing P concentrations, assuming that different P pools have distinct δ^18^O_P_ values.^3^ Neither P concentrations nor δ^18^O_P_ values were influenced by extraction temperature, presumably due to the short extraction time. In contrast, the P_unf_ and P_fum_ concentrations determined via the AEM method, which takes 16 h, were influenced by extraction temperature, with lower concentrations at 4°C than at 22°C (Table 3).

For Madden Dam, increasing the fumigation time reduced the δ^18^O_P_ values of Bray‐1 P_fum_ slightly, but not significantly. The differences between the δ^18^O_P_ values of P_unf_ and P_fum_ by Bray‐1 were small, which makes it difficult to accurately calculate the δ^18^O_P_ values of microbial P. It is most likely that at this site the δ^18^O_P_ values of microbial P and P_unf_ are similar. For Plantation Road, the concentrations of Bray‐1 P_unf_ were around the lower limit of the purification method and we could not obtain a sufficient amount of silver phosphate for the δ^18^O_P_ determination. We would have needed at least 100 g of fresh soil to yield a sufficient amount of P. This is still an order of magnitude less than the 1 kg of fresh soil needed in the case of the AEM method, but would require the volume of the Bray‐1 extract to be reduced, for example by using the MAGIC method.^37^ For Plantation Road, the δ^18^O_P_ values of Bray‐1 P_fum_ did not change with fumigation time, but nor did the Bray‐1 P_fum_ concentrations. Consequently, the contribution of microbial P to Bray‐1 P_fum_ might be too small to detect in the δ^18^O_P_ values of Bray‐1 P_fum_ or, as for Madden Dam, the δ^18^O_P_ values of microbial P and P_unf_ were similar.

### 5.2 δ^18^O_P_ values in Bray‐1 and AEM extracts

The Bray‐1 P_unf_ (20.7‰ for Madden Dam, average of samples extracted at 4°C using different solution‐to‐soil ratios) were more enriched in^18^O than AEM P_unf_ (18.0‰ Madden Dam, extracted at 4°C). The AEM method takes longer than the Bray‐1 method, so the possibility cannot be excluded that some microbial P (composed of organic and inorganic P) is released during the AEM extraction of unfumigated samples. Release of inorganic P from microbial cells would not be detected using^18^O‐labelled and unlabelled solutions as no O exchange occurs, but it would reduce the δ^18^O_P_ values in our soils because the δ^18^O_P_ values of microbial P are probably lower than the values for available P based on the results for AEM P_unf_ and P_fum_. If we assume a δ^18^O_P_ value of microbial P of 12‰ for Madden Dam (calculated using the δ^18^O_P_ values of AEM P_unf_ and P_fum_ and the corresponding concentrations) based on δ^18^O_P_ values, 40% of the AEM P_unf_ would need to come from inorganic microbial P and this seems unlikely. Hydrolysis of organic P during the extraction could also release inorganic P with relatively low δ^18^O_P_ values based on our experimental conditions (i.e. extraction temperature for AEM 4°C and a δ^18^O value of the water used for the extraction of −4.2‰), but this also seems unlikely. It is possible that the Bray‐1 solution extracts a different pool of inorganic P from that extracted by AEM, with different δ^18^O_P_ values,^3^ which would not be detected using^18^O‐labelled and unlabelled solutions. Thus, the differences between the δ^18^O_P_ values of AEM and Bray‐1 P_unf_ might be explained by a combination of differences in P pools and changes during extraction. Given that the Bray‐1 method seems less likely to be influenced by extraction artefacts (release of inorganic and organic P from microorganisms) than the AEM method due to the shorter extraction time, it should provide a more accurate measure of the available P in the soil.

The δ^18^O_P_ value of microbial P calculated based on the Bray‐1 method differed markedly from the δ^18^O_P_ value of microbial P calculated based on the AEM method. The concentrations of Bray‐1 P_fum_ were lower than the concentrations of AEM P_fum_. The same was true for Bray‐1 P_unf_ compared with AEM P_unf_, but the δ^18^O_P_ values were closer. It is possible that chloroform fumigation was less efficient than hexanol fumigation, but we have no evidence for this. Phosphate released during the 24 h chloroform fumigation can be re‐adsorbed onto the soil. Sorption/desorption only has a minor effect on the δ^18^O_P_ values, leading to a depletion in^18^O of the sorbed phosphate, but this is only apparent at the beginning of a sorption/desorption experiment.^38^ However, the δ^18^O_P_ values of Bray‐1 P_fum_ changed only slightly with fumigation time for Madden Dam soil and did not change for Plantation Road soil. It is thus unlikely that sorption/desorption caused the differences in δ^18^O_P_ values between the Bray‐1 and AEM method, and this requires further investigation. One possibility would be to use a wider solution‐to‐soil ratio, i.e. up to 20, for the determination of the δ^18^O_P_ values of Bray‐1 P_fum_, as our results showed that the P concentrations in Bray‐1 extracts increased with increasing solution‐to‐soil ratio, but only to a certain threshold (Figure 1).

## 6 CONCLUSIONS

The Bray‐1 method has advantages over the AEM method for the determination of the δ^18^O_P_ values of available P. Bray extraction is rapid and therefore has a higher sample throughput, does not require cold temperatures, uses a relatively small mass of soil, and minimizes the possibility of artefacts (e.g. lysis of microbial cells, continual exchange of P with the solid phase) impacting δ^18^O_P_ values. In addition, Bray extraction is robust, because variations in extraction conditions (e.g. soil‐to‐solution ratio) do not influence δ^18^O_P_ values. However, further investigation of the difference between the δ^18^O_P_ values of microbial P Bray‐1and those of microbial P AEM is required to identify the most accurate way to determine the δ^18^O_P_ value of microbial P. The advantage of the Bray‐1 method is its rapid extraction time, although more microbial P is extracted using the AEM method. Overall, the Bray‐1 method provides a suitable alternative procedure for determining the δ^18^O_P_ values of available P for strongly weathered tropical forest soils. Given the advantages of the procedure, it seems likely to also have application for acidic soils in a variety of ecosystems worldwide.
